# Measuring causes of adult mortality in rural northern Malawi over a decade of change

**DOI:** 10.3402/gha.v7.23621

**Published:** 2014-04-30

**Authors:** Judith R. Glynn, Clara Calvert, Alison Price, Menard Chihana, Lackson Kachiwanda, Sebastian Mboma, Basia Zaba, Amelia C. Crampin

**Affiliations:** 1 London School of Hygiene and Tropical Medicine, University of London, London, UK; 2 Karonga Prevention Study, Chilumba, Malawi

**Keywords:** HIV, mortality, verbal autopsy, antiretroviral therapy, Africa

## Abstract

**Background:**

Verbal autopsy could be more widely used if interpretation by computer algorithm could be relied on. We assessed how InterVA-4 results compared with clinician review in diagnosing HIV/AIDS-related deaths over the period of antiretroviral (ART) roll-out.

**Design:**

In the Karonga Prevention Study demographic surveillance site in northern Malawi, all deaths are followed by verbal autopsy using a semi-structured questionnaire. Cause of death is assigned by two clinicians with a third as a tie-breaker. The clinician review diagnosis was compared with the InterVA diagnosis using the same questionnaire data, including all adult deaths from late 2002 to 2012. For both methods data on HIV status were used. ART was first available in the district from 2005, and within the demographic surveillance area from 2006.

**Results:**

There were 1,637 adult deaths, with verbal autopsy data for 1,615. Adult mortality and the proportion of deaths attributable to HIV/AIDS fell dramatically following ART introduction, but for each year the proportion attributed to HIV/AIDS by InterVA was lower than that attributed by clinician review. This was partly explained by the handling of TB cases. Using clinician review as the best available ‘gold standard’, for those aged 15–59, the sensitivity of InterVA for HIV/AIDS deaths was 59% and specificity 88%. Grouping HIV/AIDS/TB sensitivity was 78% and specificity 83%. Sensitivity was lower after widespread ART use.

**Conclusions:**

InterVA underestimates the proportion of deaths due to HIV/AIDS. Accepting that it is unrealistic to try and differentiate TB and AIDS deaths would improve the estimates. Caution is needed in interpreting trends in causes of death as ART use may affect the performance of the algorithm.

Over the past 20 years, there have been dramatic changes in both the levels and causes of mortality in sub-Saharan Africa. Initial catastrophic rises in mortality due to HIV have been partially curtailed by antiretroviral therapy (ART) (1), and deaths due to diseases conventionally associated with more affluent societies are becoming more prominent (2).

Few countries in sub-Saharan Africa have vital registration or death certification, so estimation of mortality rates and distribution of causes of death relies on indirect methods or on detailed studies in demographic surveillance sites. In these sites, regular population updates through repeated censuses and/or continuous registration systems ensure almost complete registration of births and deaths. Accurate determination of the cause of death is much more complex. Many deaths will occur without clinical care, and diagnostic facilities for those who do reach care are often limited.

Demographic surveillance sites rely on verbal autopsy to classify major causes of death (3, 4). An informant, preferably the caregiver during the final illness, is interviewed using a set of standard questions. Information may be added from health passports if available. A diagnosis is made based on clinician review or through computer algorithms such as InterVA (5). In another paper in this series, the performance of InterVA-4 based on responses only (not HIV status unless reported during the verbal autopsy) was assessed against known HIV status across six demographic surveillance sites (6). This showed a high specificity (90%) but could not estimate sensitivity as HIV-positive individuals can die from other causes.

The Karonga Prevention Study, one of those six sites, has unusually detailed data collection and clinician review (7). We have previously documented adult mortality rates and causes of death up to 2009, showing a decline in all-cause mortality following antiretroviral roll-out and replacement of HIV/AIDS by non-communicable disease as the leading cause of death (2). We now update this analysis to 2012 and assess the performance of InterVA against the conclusions reached by the clinicians and investigate how this varies by age group, sex, time period (pre- and post-antiretroviral roll-out), and HIV status.

## Methods

The demographic surveillance site covers a population of about 35,000 in the southern part of Karonga District, northern Malawi. It is predominantly rural, although half of the population lives within 1 km of a tarmac road. The main occupation is subsistence farming. HIV was already present in the area in the early 1980s. The prevalence peaked in the mid-1990s and is now around 9% in women and 7% in men (8). The HIV is mainly subtype C (9).

Demographic surveillance started in 2002, with a baseline house-to-house census which covered the whole area by 2004 (10). This records detailed data on personal identifiers, sociodemographic and economic status for all individuals, and the physical location of all households. Community-based key informants are responsible for updating information within defined geographical areas that cover between 30 and 40 households. They report monthly on births and deaths and collect migration data for annual reports in their area. Births and deaths are followed-up immediately by the field staff, and all events are checked during annual re-censuses.

In 2005–06, a HIV sero-survey was conducted in selected clusters of the demographic surveillance area. Between 2007 and 2011, four annual house-to-house HIV sero-surveys were completed in the whole demographic surveillance area. HIV rapid tests were used, with results available to the participants during the visit (11). The sero-surveys followed about 6 weeks after re-census in each area (8). All adults (≥15 years old) who were resident in the demographic surveillance area at the time of the re-census were visited at home, with up to three repeat visits if necessary. Consent was sought separately for interviewing and HIV testing. Participants were encouraged to know their status, but could accept testing and choose not to know the result, in which case HIV testing was performed at project headquarters. Interviewers asked about previous HIV testing, including the timing and result of the most recent test, and about ART use if the participant reported that they were HIV positive. HIV-positive individuals who elected to know their status were referred for screening for eligibility for ART. HIV results are also available from linked epidemiological studies within the area.

Free ART was first available at the Karonga district hospital in July 2005, in the rural hospital within the demographic surveillance area in September 2006, and at further clinics within the area from October 2010. By mid-2008, ART uptake in the demographic surveillance area was estimated to be 58% of those eligible (12). Initial criteria for eligibility were WHO stage 3 or 4 disease or CD4 of <250 cells/mm^3^ (after a brief period of <200). CD4 counts were rarely available so eligibility was usually assessed clinically. The criteria for eligibility widened to include those with CD4 up to 350 cells/mm^3^ in July 2011.

### Cause of death

After a death has been reported by a key informant, a verbal autopsy is conducted as soon as possible, after allowing a 2-week mourning period. An interviewer with clinical training visits the deceased's household, and if consent is given, fills in a semi-structured verbal autopsy form (2). This is similar to the 2003 INDEPTH verbal autopsy tool, which was an adaptation of the contemporaneous WHO questionnaire (13). Close relatives of the deceased, preferably those who were present most of the time or who nursed the deceased before they died, are asked an open-ended narrative question about the death, and then a series of closed questions about specific symptoms. The completed questionnaire is given to two independent clinicians (physicians or clinical officers) who independently assign a cause of death. If the two assigned causes of death are discordant, a third physician reviewer assesses all the evidence, including the two previous reviews, and assigns a cause of death. Information on HIV status is available to the reviewers if known. Data on ART use was asked specifically from 2009 and may have been mentioned by relatives before that. Deaths of individuals with tuberculosis were assigned as ‘TB’, if they had good evidence of tuberculosis (TB) and no other symptoms of AIDS. Otherwise, they were classified as ‘AIDS’ or ‘TB/AIDS unspecifiable’. Different clinicians have assigned the cause of death over the long period of study. The two coding clinicians agreed on cause of death (comparing the categories ‘not TB/AIDS’, ‘AIDS’, ‘TB’, ‘TB or AIDS’, ‘unspecifiable’) for 82% of deaths (Kappa 0.68). Grouping the TB and AIDS categories, the overall agreement increased to 87%, Kappa 0.76.

For the InterVA analysis, the original data from the verbal autopsy forms were entered according to the InterVA-4 required format. Data were directly extracted from the questionnaire for 112 of the 190 InterVA-4 input items which are relevant for adult deaths. The remaining input items were left as missing. Data were available for all the key symptoms associated with HIV-related deaths (e.g. oral candidiasis and wasting), and where possible, available data on HIV status, including self-report, were also included in the InterVA input.

### Ethical approval

Ethical approval for the study was obtained from the National Health Sciences Research Committee of Malawi and the Ethics Committee of the London School of Hygiene and Tropical Medicine.

### Data analysis

Data from August 2002 to the end of 2012 were included. The study period was divided into three: pre ART (before 7 July 2005); during ART-rollout up to 2 years after ART availability within the demographic surveillance area (7 July 2005–6 September 2008); and more than 2 years after the availability of ART locally (after 6 September 2008). Person-time at risk (in years) was used as the denominator in the calculation of all mortality rates. Analysis was done using Stata version 13 and the InterVA-4 Model was run through R Studio (version 1.0) (14). InterVA-4 requires estimates of the prevalence of HIV and malaria among deaths in the population; these were set to be high (>1% of all deaths).

Analyses compared the overall mortality rates and the proportion of deaths attributed to HIV/AIDS as estimated by InterVA and by clinician review. We used the InterVA estimations [as in Ref. (6)] but included any additional available HIV data not already reported during the verbal autopsy. InterVA calculates the likelihood for each cause of death for each individual, and these are summed across the whole population or subgroups. We explored the effect of including or excluding TB cases with AIDS cases in both the InterVA and clinician estimates as the symptoms and signs of TB and AIDS are difficult to distinguish. Finally, we compared the most likely cause of death for each individual as assigned by InterVA with that assigned by clinician review. While clinician review is far from perfect, it is the best available diagnosis, and was used as a ‘gold standard’ against which to estimate the sensitivity and specificity of InterVA. The data used by the two methods was almost the same, except that the clinicians see the narrative which is not available to InterVA, and InterVA was given additional data on HIV status (from self-report and ART use data) that was not necessarily known to the clinicians unless mentioned in the narrative.

## Results

From August 2002 to December 2012, a total of 1,637 deaths of individuals aged 15 years and above were registered in 155,875 person-years at risk giving a crude mortality rate of 10.5/1,000 person-years [95% confidence interval (CI): 10.0–11.0]. Verbal autopsy questionnaire data were available for 1,618 (99%). Three had no clinician review available, leaving 1,615 with information from both methods.

Table 1 shows age-specific all-cause mortality rates stratified by ART availability. All cause adult mortality rates declined from 13.8/1,000 person-years (95% CI: 12.6–15.1) before ART introduction in the district to 8.5 (95% CI: 7.9–9.2) once ART provision was widespread, with the reduction seen in all age groups except the over 60s.

**Table 1:** Age-specific mortality rates per 1,000 person years (all causes, both sexes) by ART availability

	Before ART introduction (before 7 July 2005)			ART introduction (7 July 2005–6 September 2008)			>2 years local ART (after 6 September 2008)		
			
Age group	Number of deaths	Number of person years	Mortality rate (95% CI)	Number of deaths	Number of person years	Mortality rate (95% CI)	Number of deaths	Number of person years	Mortality rate (95% CI)
15–19	11	6,277	1.75 (0.97–3.16)	11	10,117	1.09 (0.60–1.96)	15	13,822	1.09 (0.65–1.80)
20–24	21	5,702	3.68 (2.40–5.65)	17	9,212	1.85 (1.15–2.97)	31	10,626	2.92 (2.05–4.15)
25–29	28	4,568	6.13 (4.23–8.88)	41	7,676	5.34 (3.93–7.25)	31	9,754	3.18 (2.24–4.52)
30–34	50	3,373	14.82 (11.23–19.56)	72	6,203	11.61 (9.21–14.62)	30	8,242	3.64 (2.55–5.21)
35–39	49	2,567	19.09 (14.43–25.25)	59	4,283	13.77 (10.67–17.78)	45	6,554	6.87 (5.13–9.20)
40–44	50	2,016	24.80 (18.80–32.72)	46	3,636	12.65 (9.48–16.89)	43	4,650	9.25 (6.86–12.47)
45–49	33	1,670	19.76 (14.05–27.79)	41	2,721	15.07 (11.09–20.46)	41	3,903	10.50 (7.73–14.27)
50–54	21	1,172	17.91 (11.68–27.48)	35	2,191	15.98 (11.47–22.25)	30	3,013	9.96 (6.96–14.24)
55–59	28	1,220	22.96 (15.85–33.25)	36	1,625	22.15 (15.98–30.7)	31	2,359	13.14 (9.24–18.68)
60 +	150	3,451	43.47 (37.04–51.01)	239	5,861	40.78 (35.93–46.29)	302	7,412	40.75 (36.40–45.61)
Overall	441	32,015	13.77 (12.55–15.12)	597	53,523	11.15 (10.29–12.09)	599	70,334	8.52 (7.86–9.23)

The proportion of deaths attributable to HIV/AIDS in different time periods according to the different methods of ascribing cause is shown in Table 2 for those aged 15–59 and in Table 3 for those aged 60 and over. HIV status was known (by test result or report) for 606 deaths, of which 272 were HIV positive. One hundred and seventy of these 272 HIV-positive deaths were reported as HIV positive as part of the verbal autopsy, and 286/334 HIV negatives were reported as negative.

**Table 2:** Percentage of deaths attributable to HIV/AIDS using different methods of interpreting the verbal autopsy data in individuals aged 15–59

		Percentage of deaths attributed to HIV/AIDS			
		
Calendar year	Number of deaths	InterVA (not including TB)	InterVA (including TB)	Clinician review (excluding deaths attributed only to TB)	Clinician review (including all TB deaths)
2002/2003	96	39.6	56.1	67.7	68.8
2004	135	32.9	49.6	59.3	61.5
2005	120	50.5	60.4	60.8	61.7
2006	124	45.1	56.8	50.8	55.7
2007	99	33.9	50.5	41.4	44.4
2008	95	26.6	39.1	36.8	41.1
2009	69	21.8	31.3	40.6	44.9
2010	49	27.3	33.0	38.8	38.8
2011	69	22.3	32.3	30.4	34.8
2012	80	18.1	33.3	30.0	31.3
Overall	936	33.8	46.8	48.0	50.6

**Table 3:** Percentage of deaths attributable to HIV/AIDS using different methods of interpreting the verbal autopsy data in individuals aged 60 and over

		Percentage of deaths attributed to HIV/AIDS			
		
Calendar year	Number of deaths	InterVA (not including TB)	InterVA (including TB)	Clinician review (excluding deaths attributed only to TB)	Clinician review (including all TB deaths)
2002/2003	33	9.2	20.3	9.1	12.1
2004	70	4.5	15.2	5.7	8.6
2005	84	7.6	14.4	7.1	9.5
2006	71	7.2	11.6	8.5	8.5
2007	74	7.6	15.6	2.7	4.1
2008	76	3.9	7.9	4.0	6.6
2009	75	1.2	13.8	1.3	1.3
2010	71	0.4	9.3	1.4	2.8
2011	58	1.1	6.1	3.5	6.9
2012	67	0	10.5	1.5	6.0
Overall	679	4.2	12.2	4.3	6.3

For those aged 15–59, the proportion of deaths due to HIV/AIDS decreased over time whichever method was used, but the proportion attributed to HIV/AIDS was higher when based on clinician review than when using InterVA. This was partially explained by the way TB was handled by the two methods. If TB deaths were grouped with AIDS deaths, there was less discrepancy between the methods. In those aged 60 and over, the proportion of deaths attributable to HIV/AIDS was much lower, and similar in InterVA and clinician review. In this age group, including TB deaths greatly increased the discrepancy, with InterVA over-diagnosing TB deaths compared to the clinician review.

To allow a more detailed examination of the diagnoses reached by InterVA and clinician review, the most likely cause determined by InterVA for each individual was compared with that from the clinician review (Table 4). This shows the results by age group, and for those aged 15–59, by sex, time period, and HIV status. Of the 320 deaths in age group 15–59 attributed to HIV/AIDS by InterVA, clinician review attributed 259 to AIDS or TB/AIDS, 2 to TB only, 6 to other communicable disease, 40 to non-communicable disease, 1 to external causes, and 12 were indeterminate. Of the 124 attributed to TB by InterVA, only 18 were attributed to TB alone by clinician review with 11 attributed to TB/AIDS, 75 to AIDS, 2 to other communicable disease, and 15 to non-communicable disease. By contrast there was poor correlation between InterVA and clinician diagnoses for the over 60s. Of 30 deaths attributed to HIV/AIDS by InterVA, 9 were attributed to AIDS or TB/AIDS by clinician review, with 6 other communicable diseases and 14 non-communicable diseases. And of 56 attributed to TB by InterVA, clinician review attributed 12 to TB or TB/AIDS, 7 to AIDS, 3 to other communicable disease, and 32 to non-communicable disease.

**Table 4:** Comparison of InterVA to causes of death given through clinician review

			Clinician review							
			
		Main cause of death assigned by InterVA	AIDS death only	TB/AIDS death	TB death only	Other communicable	Non-communicable	Maternal/External	Indeterminate	Total
Overall (age 15–59)		HIV/AIDS	251	8	2	6	40	1	12	320
		TB	75	11	18	2	15	1	2	124
		Other	96	2	5	84	129	105	26	447
		Indeterminate	6	0	0	3	14	10	12	45
		Total	428	21	25	95	198	117	52	936
Age group	15–29	HIV/AIDS	40	3	2	2	10	0	1	58
		TB	7	2	2	1	3	0	0	15
		Other	14	0	0	21	25	55	5	120
		Indeterminate	0	0	0	0	4	2	3	9
		Total	61	5	4	24	42	57	9	202
	30–44	HIV/AIDS	141	4	0	2	20	1	9	177
		TB	47	7	9	0	6	1	2	72
		Other	49	2	4	36	38	35	13	177
		Indeterminate	2	0	0	1	4	5	4	16
		Total	239	13	13	39	68	42	28	442
	45–59	HIV/AIDS	70	1	0	2	10	0	2	85
		TB	21	2	7	1	6	0	0	37
		Other	33	0	1	27	66	15	8	150
		Indeterminate	4	0	0	2	6	3	5	20
		Total	128	3	8	32	88	18	15	292
	60 and over	HIV/AIDS	9	0	0	6	14	0	1	30
		TB	7	2	10	3	32	0	2	56
		Other	9	1	4	81	365	19	66	545
		Indeterminate	1	0	0	5	11	2	29	48
		Total	26	3	14	95	422	21	98	679
Sex (age 15–59)	Men	HIV/AIDS	107	5	0	3	19	0	8	142
		TB	37	5	13	1	8	0	1	65
		Other	49	1	4	57	63	52	16	242
		Indeterminate	4	0	0	1	11	8	11	35
		Total	197	11	17	62	101	60	36	484
	Women	HIV/AIDS	144	3	2	3	21	1	4	178
		TB	38	6	5	1	7	1	1	59
		Other	47	1	1	27	66	53	10	205
		Indeterminate	2	0	0	2	3	2	1	10
		Total	231	10	8	33	97	57	16	452
Time period	Before ART introduction	HIV/AIDS	95	4	0	2	5	0	3	109
		TB	29	9	2	1	4	0	0	45
(age 15–59)		Other	40	2	3	20	28	19	13	125
		Indeterminate	2	0	0	0	3	5	1	11
		Total	166	15	5	23	40	24	17	290
	ART introduction	HIV/AIDS	115	4	2	2	20	1	4	148
		TB	30	0	10	1	4	1	1	47
		Other	23	0	1	38	47	34	7	150
		Indeterminate	1	0	0	1	4	3	4	13
		Total	169	4	13	42	75	39	16	358
	>2 years local ART	HIV/AIDS	41	0	0	2	15	0	5	63
		TB	16	2	6	0	7	0	1	32
		Other	33	0	1	26	54	52	6	172
		Indeterminate	3	0	0	2	7	2	7	21
		Total	93	2	7	30	83	54	19	288
Known HIV status (age 15–59)	HIV negative	HIV/AIDS	1	0	0	2	12	0	0	15
		TB	0	0	7	0	9	0	0	16
		Other	0	0	0	15	46	49	5	115
		Indeterminate	0	0	0	1	5	3	7	16
		Total	1	0	7	18	72	52	12	162
	HIV positive	HIV/AIDS	104	4	1	1	8	0	3	121
		TB	37	4	6	0	0	0	2	49
		Other	49	1	1	12	9	6	2	80
		Indeterminate	4	0	0	0	1	0	0	5
		Total	194	9	8	13	18	6	7	255
	HIV unknown	HIV/AIDS	146	4	1	3	20	1	9	184
		TB	38	7	5	2	6	1	0	59
		Other	47	1	4	57	74	50	19	252
		Indeterminate	2	0	0	2	8	7	5	24
		Total	233	12	10	64	108	59	33	519
	On ART	HIV/AIDS	37	2	0	0	3	0	1	43
		TB	11	0	2	0	0	0	1	14
		Other	16	0	0	4	1	1	0	22
		Indeterminate	2	0	0	0	0	0	0	2
		Total	66	2	2	4	4	1	2	81

Diagnoses reached by InterVA and clinician review were also compared by HIV status (Table 4). Neither method assigned HIV/AIDS as the cause if there was a recent negative HIV test (with one exception) and neither method automatically assigned HIV/AIDS as the cause if there was a positive HIV test. The relative under-reporting of HIV/AIDS deaths by InterVA was more marked in those known to be HIV positive than HIV unknown: among those known to be HIV positive, HIV/AIDS deaths were diagnosed for 47% by InterVA and 80% by clinician review (53 and 84%, respectively, if on ART). For those with unknown status, HIV/AIDS deaths were diagnosed for 35% by InterVA and 47% by clinician review.

The sensitivity and specificity of InterVA compared to clinician review is shown in Table 5 (excluding those with undetermined cause of death). Among those aged 15–59, the sensitivity of InterVA for identifying HIV/AIDS deaths was 59% and the specificity was 88%. Sensitivity decreased with age and was only 32% in those aged over 60. Among those aged 15–59, the sensitivity was lower in the last period, after ART use was widely established. Specificity varied less and was lowest during ART introduction. Grouping HIV/AIDS and TB together greatly improved the sensitivity of InterVA in all groups, with little loss in specificity. In the 15–59 age group, the sensitivity was 78% and the specificity 83% for the combined definition.

**Table 5:** Sensitivity and specificity of InterVA assignment of HIV/AIDS as the main cause of death compared with clinician review (excluding indeterminates)

		Excluding TB		Including TB	
			
		Sensitivity	Specificity	Sensitivity	Specificity
Overall (15–59 years old)		58.5	88.0	78.0	83.0
Age	15–29	65.2	88.4	80.0	86.3
	30–44	58.0	84.9	79.1	78.4
	45–59	55.9	91.1	74.8	85.0
	60 and over	32.1	96.3	66.7	89.4
Sex (15–59 years old)	Male	54.9	90.0	75.6	84.7
	Female	61.5	85.6	80.2	81.1
Time period (15–59 years old)	Before ART introduction	55.3	91.7	75.5	84.8
	ART introduction	69.2	84.5	87.0	80.4
	>2 years local ART	44.6	89.6	65.7	84.6

## Discussion

In this setting, adult mortality rates have fallen dramatically since ART became widely available. This is mirrored by the decreasing proportion of deaths attributable to HIV/AIDS. This decrease is seen with both methods of classifying the verbal autopsy data – InterVA or clinician review – but there were differences between them, with the proportion attributed to HIV/AIDS by InterVA being lower than that attributed by clinician review in all year groups for those aged 15–59. The difference between the two methods was particularly marked in those who were known to be HIV positive.

Part of the difference in performance in the 15–59 age group is explained by the classification of TB and TB/AIDS deaths. If all TB deaths were grouped with the AIDS deaths for both methods the results were much more similar, although clinician review continued to attribute a slightly higher proportion to HIV/AIDS/TB in this age group.

In the over 60s, the proportion of deaths attributed to HIV/AIDS without TB was low and similar in the two methods. In this age group, the inclusion of TB deaths greatly increased the proportion attributed to HIV/AIDS/TB by InterVA, with much less increase in the clinician review diagnoses. As shown in Table 4, of the 56 TB cases diagnosed by InterVA in the over 60s, only 34% (19 cases) were classified as TB or AIDS by clinician review, and 57% (32 cases) were thought to be due to non-communicable disease. By contrast, among those aged 15–59 diagnosed as TB deaths by InterVA, 84% (104/124) were classified as TB or AIDS, and only 12% (15/124) as non-communicable disease by clinician review. In Karonga, TB case finding and diagnosis is good, as TB has been a major focus of studies for 30 years, so it is likely that it is the InterVA diagnoses that are more often incorrect. In the over 60s, although the overall numbers attributed to HIV/AIDS by the two methods was similar, at an individual level there was poor correlation so the similarity is probably due to chance.

The estimates of sensitivity and specificity show that the sensitivity of InterVA can be greatly improved, with little loss of specificity, by grouping HIV/AIDS/TB as one diagnostic category. An analysis in Nairobi comparing InterVA with AIDS mortality estimates from spectrum also found under-estimation by InterVA which was improved by including deaths classified as TB (15). A study in Ethiopia using hospital diagnoses and HIV testing as the comparison group found improved sensitivity without loss of specificity for InterVA when combining TB and AIDS (16), and a study in Kenya found that InterVA tended to over-diagnose TB when compared to hospital diagnoses (17). A comparison in a demographic surveillance site in Ethiopia found poor agreement between InterVA and physician review for both HIV/AIDS and TB diagnoses (18), and worse agreement in older adults (19). A large multi-country study also found relatively poor performance by InterVA compared to physician review where tertiary hospital cause of death assignments, with stringent diagnostic criteria, were used as the gold standard (20, 21), though methodological questions have been raised about some of the comparisons in this study (22).

Obviously, clinician review of verbal autopsy data is not a true gold standard. Even pre-mortem clinical diagnoses in settings with good clinical facilities are often contradicted by conventional autopsy (3, 23, 24), and reaching a diagnosis based on a limited amount of reported information is much more difficult. What the comparison does test, however, is how well the computer algorithm performs compared to two (or in cases of discrepancy, three) clinicians working in the local environment, with essentially the same information. The results suggest that using the InterVA definition of HIV/AIDS alone will underestimate AIDS death, but that it performs better for the combined diagnosis of HIV/AIDS/TB, and this amendment could be recommended in the 15–59 age group. Results in the over 60s appear less reliable. The apparently lower sensitivity of InterVA for diagnosing HIV/AIDS/TB after established ART use in the population suggests that considerable caution will be needed in interpreting trends in HIV-related deaths.
